# Nitric oxide signaling pathway activation inhibits the immune escape of pancreatic carcinoma cells

**DOI:** 10.3892/ol.2014.2607

**Published:** 2014-10-10

**Authors:** YEBIN LU, JUANJUAN HU, WEIJIA SUN, XIAOHUI DUAN, XIONG CHEN

**Affiliations:** Department of General Surgery, Xiangya Hospital, Central South University, Changsha, Hunan 410008, P.R. China

**Keywords:** nitric oxide signaling pathway, pancreatic carcinoma cells, glyceryl trinitrate, immune escape

## Abstract

The aim of the present study was to investigate the effect of the nitric oxide signaling pathway on immune escape; thus, a tumorigenesis model was established using nude mice. The mice were inoculated with pancreatic carcinoma cells and divided into two groups, a glyceryl trinitrate (GTN) and a placebo group. When tumor volumes reached 150 mm^3^, the mice in the GTN group were treated with GTN transdermal patches (dose, 7.3 μg/h) while the mice in the placebo group were administered untreated patches. Following treatment, the tumor volume was recorded every 3–4 days and after 28 days, the tumors were analyzed. The results indicated that GTN treatment may reduce the levels of soluble major histocompatibility complex class I chain-related molecules, and natural killer group 2 member D, as well as inhibiting tumor growth.

## Introduction

A complex process for the immune system is preserving the integrity of the ‘self’, while protecting the ‘self from ‘non-self’ and dangerous invaders (1). Tumors, which are derived from the ‘self’, exhibit a high proliferative potential and present a marked risk to the host. In addition, due to the high mutation rate, it is difficult for the immune system to respond; therefore, immune escape may occur.

On identification of ‘non-self’ in the body, the immune system initiates a series of responses to eliminate the ‘non-self’, during which various immune factors are synthesized, including major histocompatibility complex class I chain-related (MIC) molecules and natural killer group 2 member D (NKG2D). The MIC family consists of seven members, however, only MIC A and MIC B encode proteins (2). The MIC A/B proteins are located in the cell membrane and act as ligands for NKG2D. They are rarely expressed by normal cells, however, are broadly expressed in a variety of malignancies (3–5). NKG2D is the receptor that is expressed on natural killer (NK) cells. Following ligand binding, the receptor transfers the signal downstream to activate the cytotoxic activity of the NK cells (6–8).

Nitric oxide (NO), a highly soluble gas, is generated from L-arginine and oxygen, which is catalyzed by NO synthase (9). The generated NO activates soluble guanylyl cyclase (sGC) (10–14), which leads to the formation of cyclic guanosine monophosphate (cGMP), which in turn activates the cGMP-dependent protein kinase (PKG) and downstream effectors. Previous studies have revealed that NO inhibition of tumor growth may occur as a result of the generation of hydroxyl radicals, inactivation of enzymes involved in the respiratory chain or failure to replicate.

## Materials and methods

### Materials

TRIzol was purchased from Invitrogen Life Technologies (Carlsbad, CA, USA) and M-MLV reverse transcriptase was purchased from Promega Corporation (Madison, WI, USA). MIC A/B and NKG2D mouse anti-human monoclonal antibodies were purchased from Santa Cruz Biotechnology, Inc. (Santa Cruz, CA, USA), hypoxia-inducible factor 1-α (HIF-1α) rabbit anti-human polyclonal antibody was purchased from Boster Systems Inc. (Pleasanton, CA, USA) and glyceryl trinitrate (GTN) transdermal patches were purchased from Schwarz Pharma (Brussels, Belgium).

### Cell culture

A PANC-1 cell line was obtained from the Shanghai Institute of Biological Sciences (Shanghai, China) and maintained in a monolayer culture in Dulbecco’s modified Eagle’s medium supplemented with 20% fetal bovine serum (FBS). The study was approved by the ethics committee of Central South University (Changsha, China).

### Analysis of tumorigenicity

All mice used were handled in compliance with the guide for the care and use of laboratory animals. A total of 32 nude mice were divided into two groups; a GTN and a placebo group. The mice were subcutaneously injected with 2×10^7^ cells and tumor size was measured using a caliper. The tumor volume was calculated using the following formula: Tumor volume (mm^3^) = [(width)^2^ × length]/2. When tumors had grown to a volume of 150 mm^3^ the GTN group was treated with GTN transdermal patches (dose, 7.3 μg/h) while the mice in the placebo group were administered untreated patches.

### Immunohistochemistry

Tumor samples were fixed with 10% paraformaldehyde and embedded in paraffin. Samples were cut into sections (thickness, 4-μm) using a Leica-RM2135 rotary microtome (Leica, Mannheim, Germany) and adhered to microscope slides. The sections were dewaxed and washed three times with phosphate-buffered saline (PBS). For non-specific blocking, sections were incubated in 10% normal goat serum for 30 min at 37°C and incubated overnight with the primary polyclonal rabbit anti-human HIF-1α and monoclonal mouse anti-human MIC A/B antibodies at 4°C (Santa Cruz Biotechnology, Inc.). Following three washes with PBS, the sections were incubated in secondary mouse polyclonal anti-rabbit and goat anti-mouse antibodies conjugated to biotin and horseradish peroxidase (HRP) marked polyclonal anti-biotin for 30 min at 37°C (Santa Cruz Biotechnology, Inc.). Next, the samples were incubated in freshly prepared 3,3′-diaminobenzidine and counterstained with hematoxylin. Finally, samples were observed under an optical microscope (CX41, Olympus Corporation, Tokyo, Japan).

The positive reaction for the MIC A/B protein results in is yellow or brown granules, located in the cell membrane and/or the cytoplasm. Briefly, each slice was randomly selected and 10 were viewed under a high-power field, the following staining intensity score was used: 0, negative staining, all cancer cells without staining; 1, weak positive staining, found in <20% of cells. In a high power field the staining is markedly strong in the cells compared with the interstitial tissue; 2, strong positive staining, staining was markedly stronger in all cancer cells compared with the interstitial tissue. MIC A/B expression was subsequently divided as follows: 0–1 points, low expression group; 2 points, high expression group.

The positive result for the HIF-1α protein is the observation of brown particles in the nucleus or cytoplasm. The entire slice was viewed under a light microscope, (magnification, ×400) were randomly observed in 10 visual fields. The staining score used was as follows: (−), no staining, or <1% nuclear staining; (+), 1–10% nuclear staining and/or weak cytoplasmic staining; (+), 10–50% nuclear staining and/or distinct cytoplasmic staining; (+++): >50% nuclear staining and/or strong cytoplasmic staining. (−)/(+) Low expression; (+)/(++) high expression.

### Reverse transcription (RT)-polymerase chain reaction (PCR)

All RNAs were extracted with TRIzol. RT was conducted using 1 μg total RNA and the following oligo (dT) primers: Forward, 5′-AGGTACATCTGGATGGTCAG-3′ and reverse, 5′-TTGTCTTCATGGATCTCACA-3′ for homo-MIC A, yielding an amplified fragment of 232 bp; forward, 5′-CTTCGTTACAACCTCATGGT-3′ and reverse, 5′-ATATGAGTCAG GGTCCTCCT-3′ for homo-MIC B, yielding an amplified fragment of 227 bp; and forward, 5′-ACCACAGTCCATGCCATCAC-3′ and reverse, 5′-TCCACCACCCTGTTGCTGTA-3′ for homo-GAPDH, yielding an amplified fragment of 450 bp. A total of 40 cycles of PCR were performed at an annealing temperature of 54°C.

### Western blot analysis

Total proteins were extracted from the tumor tissues and the protein concentration was determined using the Bradford method (15). A total of 50 μg protein was loaded per lane. Following electrophoresis, all proteins were transferred to a cellulose acetate membrane (Koch Membrane Systems, Inc., Wilmington, MA, USA) and the membrane was blocked with 5% non-fat milk in PBS overnight at 4°C. The following day, the blot was incubated with a primary monoclonal mouse anti-human antibody (1:1,000; Santa Cruz Biotechnology, Inc.) for 2 h at 37°C and washed three times with PBS. Next, the blot was incubated with a HRP-conjugated goat anti-mouse secondary antibody (1:2,000; Santa Cruz Biotechnology, Inc.) for 1 h at 37°C and X-ray films were used to observe the bands of the western blot. The proteins were analysed using Scion Image version 4.0.3.2 (Scion Corporation, Frederick, MD, USA). The protein content was observed relative to that of the GAPDH control band.

### ELISA

Two non-overlapping-epitope antibodies were used. Plates were coated with the primary mouse anti-human MIC A/B antibody overnight at 4°C, blocked with 5% FBS for 2 h at 37°C and washed with PBS. Next, samples were added and incubated for 1 h at 37°C. The plates were washed with PBS and incubated with the secondary mouse anti-human antibody conjugated with HRP for 1 h at 37°C. Finally, the substrate was added and the absorbance was measured at a wavelength of 450 nm using ELISA (DU640, Beckman Coulter, Miami, FL, USA).

### Flow cytometry

Blood was collected from the eye of the mice. Antibodies conjugated with fluorochrome were added and incubated for 20 min at room temperature in the absence of visible light. Next, hemolysin was added and the samples were centrifuged (JM-6, Beckman Coulter) at 1,000 × g for 5 min to obtain the precipitates. Following three washes with PBS, the cells were resuspended with 1% paraformaldehyde and analyzed using a BD FACSCalibur^TM^ flow cytometer (BD Biosciences, Franklin Lakes, NJ, USA).

### Statistical analysis

All statistical analyses were performed using the SPSS 13.0 statistical software package (SPSS, Inc., Chicago, IL, USA). Differences between groups were compared using a single factor analysis of variance and Student’s t-test. P<0.05 was considered to indicate a statistically significant difference.

## Results

### Effect of GTN on tumor growth

**Figure 1:**
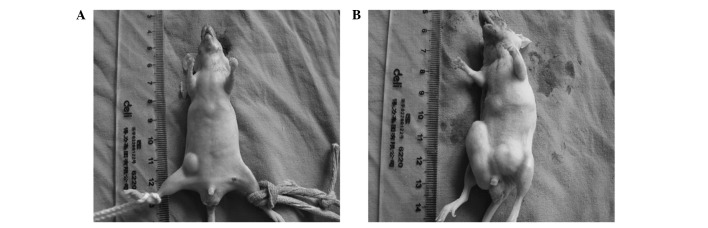
Nude mice from (A) the glyceryl trinitrate and (B) the placebo group.

**Figure 2:**
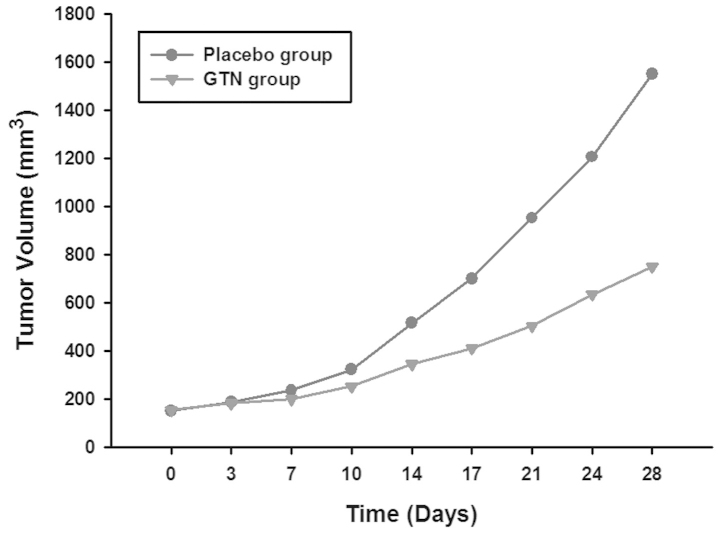
Tumor growth of the GTN and placebo groups. Tumor volume was monitored every 3–4 days. With increasing treatment time, the tumors of the GTN group were markedly smaller compared with those of the placebo group. GTN, glyceryl trinitrate.

**Figure 3:**
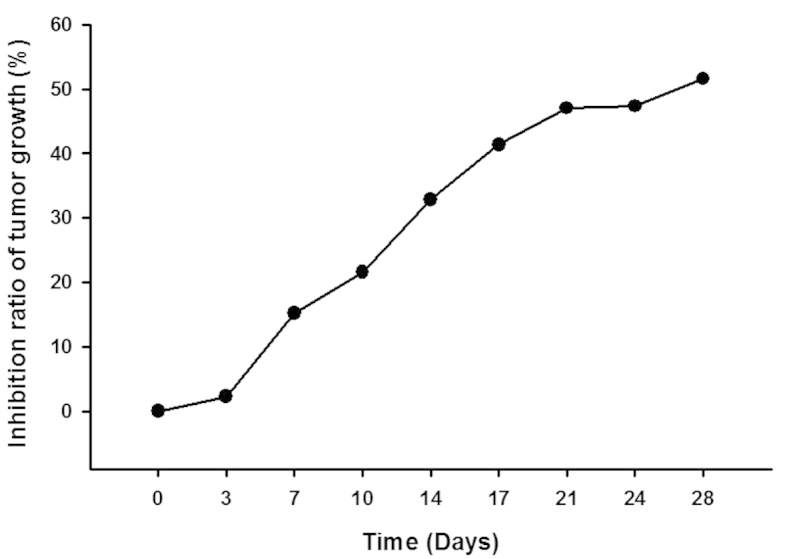
Mean inhibition ratio of tumor growth. The increase in the inhibition ratio was time-dependent.

The mice were sacrificed following 28 days of treatment. It was found that the tumors of the GTN group were markedly smaller than those of the placebo group (Figs. 1 and 2; Table I) and the inhibition ratio of tumor growth increased in a time-dependent manner (Fig. 3 and Table II).

### Effect of GTN on HIF-1α and MIC expression in tumors

**Figure 4:**
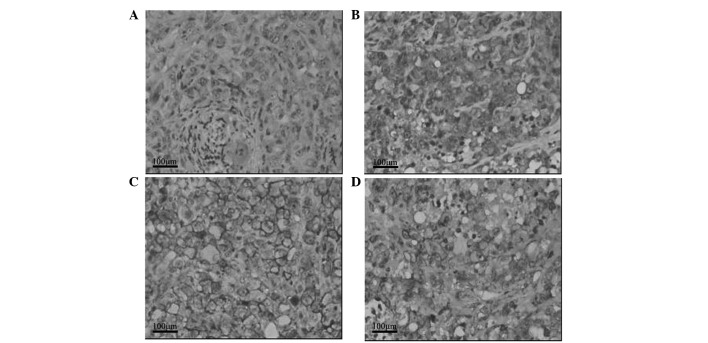
Immunohistochemical analysis of hypoxia-inducible factor-1α (HIF-1α) and major histocompatibility complex class I chain-related (MIC) expression. HIF-1α expression demonstrated in the (A) glyceryl trinitrate (GTN) and (B) placebo group. MIC expression demonstrated in the (C) GTN and (D) placebo group. Magnification, ×400.

As shown in Table III, HIF-1α expression was lower in the GTN group (8/16 mice) than that of the placebo group (14/16 mice). Notably, 12/16 mice from the GTN group highly expressed MIC when compared with the placebo group, whereby only two mice expressed MIC. Furthermore, immunohistochemical analysis revealed that HIF-1α expression was lower in the GTN group and MIC expression was higher, when compared with that of the placebo group (Fig. 4). Furthermore, HIF-1α and MIC were found to be negatively correlated (Table IV).

### Effect of GTN on MIC A/B expression in tumors

**Figure 5:**
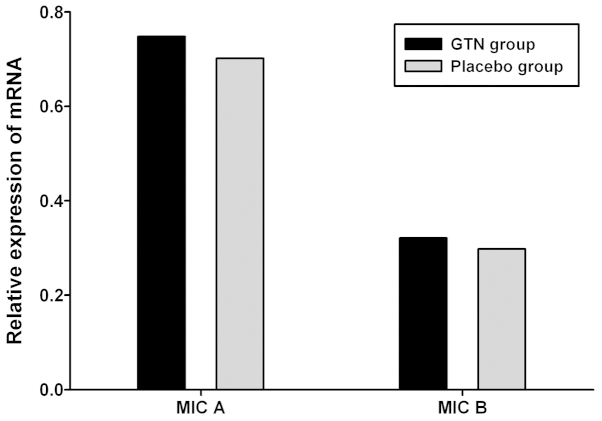
Relative expression of MIC A/B mRNA in tumors. MIC A/B, major histocompatibility class I-related chain A/B; GTN, glyceryl trinitrate.

**Figure 6:**
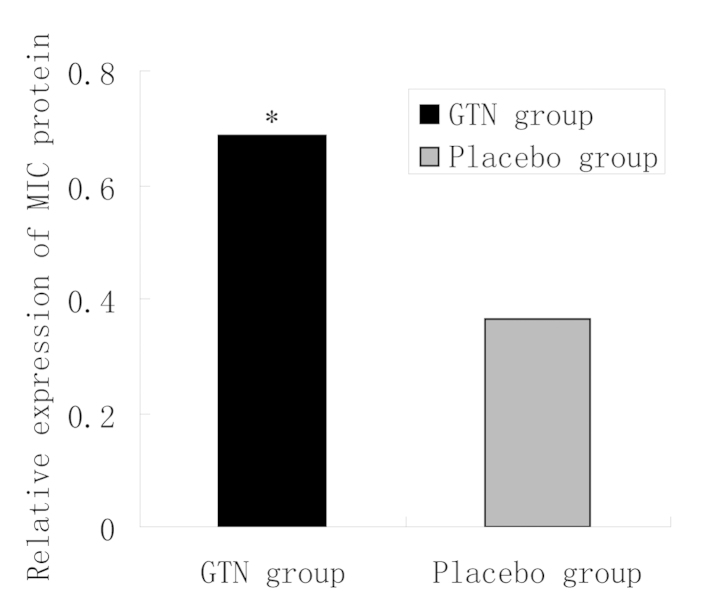
Relative expression of MIC protein in tumors. MIC, major histocompatibility complex class I chain-related; GTN, glyceryl trinitrate. ^*^P<0.01 vs. placebo group.

To investigate the effect of GTN on MIC A/B expression, total RNA and protein was extracted from the tumors. No significant difference in MIC A/B mRNA expression was identified between the GTN and placebo groups (Fig. 5 and Table V; P>0.05). However, at the protein level, MIC A/B expression was significantly higher in the GTN group compared with that of the placebo group (Fig. 6 and Table VI; P<0.01).

### Effect of GTN on soluble MIC (sMIC) in serum

**Figure 7:**
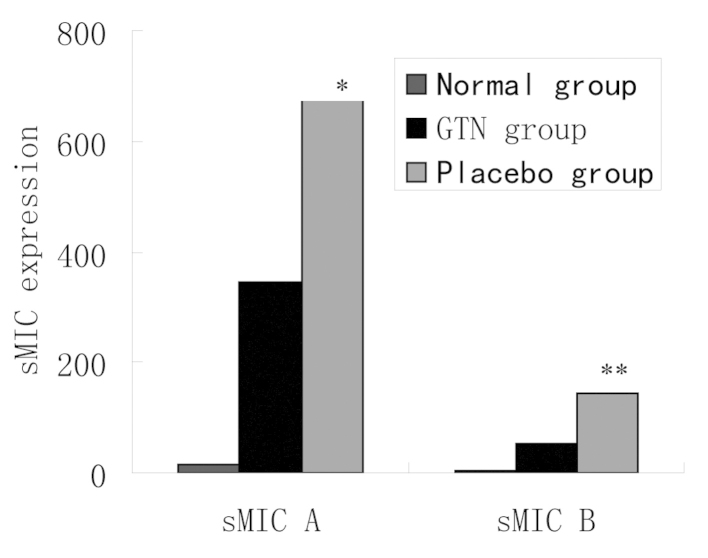
Expression of sMIC in serum.^*^P<0.001 vs. normal and GTN group. ^**^P<0.001 vs. normal and GTN group. GTN, glyceryl trinitrate; sMIC, soluble major histocompatibility complex class I chain-related.

As noted above, the same mRNA levels, but different protein levels of MIC A/B were identified between the two groups. To assess whether cellular MIC was transferred outside of the membrane, the levels of sMIC in serum were investigated. ELISA revealed that the placebo group exhibited higher levels of sMIC than the GTN group and the two groups exhibited significantly higher sMIC levels when compared with those of the normal mice (Fig. 7 and Table VII).

### Effect of GTN on NKG2D in the blood

**Figure 8:**
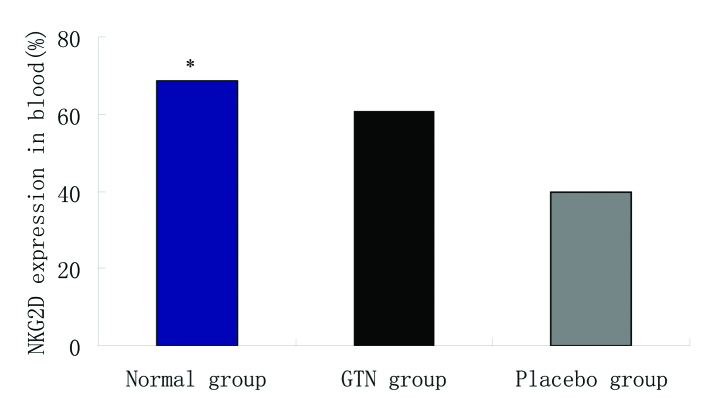
NKG2D expression in the blood. NKG2D, natural killer group 2 member D; GTN, glyceryl trinitrate. ^*^P<0.001 vs. GTN and placebo group.

**Figure 9:**
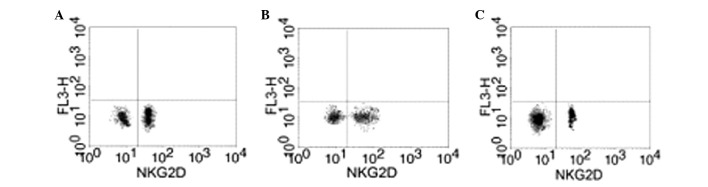
NKG2D expression in the blood of the (A) normal, (B) GTN and (C) placebo groups. NKG2D, natural killer group 2 member D; GTN, glyceryl trinitrate.

Blood samples were collected from the two groups to analyze the levels of NKG2D. Flow cytometry revealed that the GTN and normal groups exhibited significantly higher levels of NKG2D when compared with the placebo group (Figs. 8 and 9; Table VIII). In addition, NKG2D and sMIC were found to be negatively correlated (data not shown).

## Discussion

NO, synthesized by NOS, is a soluble gas which diffuses through the cell membrane and activates adenylate cyclase. Subsequently, cellular cGMP increases and acts as a second messenger to activate cGMP-dependent protein kinase G which phosphorylates downstream proteins and regulates the biological reaction, known as the NO-cGMP-PKG signaling pathway. Studies have revealed that acitivation of the NO-cGMP pathway may reverse hypoxia-induced tumor cell metastasis and chemotherapy resistance.

The formation of cancer cells causes alteration of the surface antigens to avoid immune system recognition and attack, which is termed immune escape. Activation of the NO signaling pathway may inhibit tumorigenesis. To investigate the effect of the NO signaling pathway on tumorigenesis, a nude mice model was established. All nude mice were injected with pancreatic carcinoma cells (using a PANC-1 cell line) enabling the growth of tumors. When tumor volumes reached 150 mm^3^ the GTN group were administered with GTN transdermal patches (dose, 7.3 μg/h) (16), while the placebo group were administered untreated patches. The tumor volume was then measured every 3–4 days for a period of 28 days. It was found that the tumors of the GTN group were markedly smaller when compared with those of the placebo group.

GTN, an NO donor, is commonly administered for the treatment of cardiovascular disease. Previous studies have found that GTN may also inhibit tumor cell growth, induce cell apoptosis and downregulate blood vessel- and metastasis-associated genes (17–19). GTN is firstly converted into nitrite (20–22), which reacts with glutathione and generates S-nitrosoglutathione (GSNO) (23). GSNO is subsequently decomposed and NO is released. NO is a novel cellular messenger; it activates sGC and enhances cGMP, which in turn promotes PKG as a second messenger and transfers the signal downstream (24).

NO signaling pathway activation may also inhibit immune escape. In the present study, the total RNA and protein of the tumors were extracted following 28 days of treatment and no significant difference in MIC A/B mRNA expression was identified between the GTN and placebo group. However, a significant difference was identified in the protein levels, whereby a greater level of MIC A/B expression was identified in the GTN group than in the placebo group. Furthermore, the GTN group exhibited lower levels of sMIC, but higher levels of NKG2D when compared with the placebo group. It was hypothesized that GTN does not regulate MIC expression, but suppresses the detachment of MIC from the cell membrane into the serum. Thus, sMIC levels were downregulated in the GTN group and subsequently NKG2D levels were increased, which triggered the immune system to kill the tumor cells. In addition, HIF-1α expression was identified to be lower in the GTN group compared with that in the placebo group.

In conclusion, it was hypothesized that the NO that was released from GTN enhanced the blood circulation and oxygen levels in the tumors, whilst decreasing endogenous NO synthesis and intracellular oxygen consumption. GTN activated the NO signaling pathway and also improved the hypoxic microenvironment, thus, inhibiting the immune escape of tumors cell. This study provides evidence that drugs which activate the NO pathway may present as useful treatments in cases of tumor cell metastasis.

## Figures and Tables

**Table I:** Tumor volumes for each group.

	Tumor volume ± standard deviation, mm^3^	
	
Time following treatment, days	Placebo group	Glycerol trinitrate group
0	152.3±10.3	155.0±12.6
3	187.5±20.1	183.3±16.2
7	236.3±21.2	200.5±19.4
10	323.6±35.7	253.8±26.1
14	515.9±42.0	346.3±34.1
17	700.5±74.2	410.8±46.4
21	952.2±110.3	504.3±51.5
24	1206.4±116.5	635.4±61.0
28	1550.4±148.6	750.6±71.2

**Table II:** Mean inhibition ratio of tumor growth.

Time following treatment, days	Inhibition ratio (%)
0	0
3	2.24
7	15.15
10	21.57
14	32.87
17	41.36
21	47.04
24	47.33
28	51.59

**Table III:** Expression of HIF-1α and MIC proteins in the tumors.

		HIF-1α					MIC			
			
Group	n	−	+	++	+++	P-value	0^a^	1^a^	2^a^	P-value
GTN	16	8	7	1	0	<0.001	1	3	12	0.015
Placebo	16	2	2	4	8		2	12	2	

a Staining intensity.

HIF-1α, hypoxia-inducible factor-1α; MIC, major histocompatibility complex class I chain-related; GTN, glycerol trinitrate. (−), no staining, or <1% nuclear staining; (+), 1–10% nuclear staining and/or weak cytoplasmic staining; (+), 10–50% nuclear staining and/or distinct cytoplasmic staining; (+++), >50% nuclear staining and/or strong cytoplasmic staining.

**Table IV:** Correlation between HIF-1α and MIC expression in the tumors.

	Expression			
			
Expression	MIC 0/1^a^	MIC 2^a^	r	P-value
HIF-1α			−0.601	<0.001
−/+	6	13		
++/+++	12	1		
Total	18	14		

a Staining intensity.

0, negative staining, all cancer cells without staining; 1, weak positive staining, found in <20% of cells. In a high power field the staining is markedly strong in the cells compared with the interstitial tissue; 2, strong positive staining, staining was markedly stronger in all cancer cells compared with the interstitial tissue. HIF-1α, hypoxia-inducible factor-1α; MIC, major histocompatibility complex class I chain-related.

**Table V:** Relative expression of MIC A/B mRNA in the tumors.

Group	n	MIC A, mean ±SD	t-value	P-value	MIC B, mean ± SD	t-value	P-value
GTN	16	0.748±0.112	0.402	>0.05	0.321±0.131	0.183	>0.05
Placebo	16	0.702±0.157			0.298±0.173		

GTN, glyceryl trinitrate; MIC A/B, major histocompatibility complex class I-related chain A/B; SD, standard deviation.

**Table VI:** Relative expression of the MIC protein in the tumors.

Group	n	MIC, mean ± standard deviation	t-value	P-value
GTN	16	0.688±0.214	3.969	<0.01
Placebo	16	0.363±0.123		

MIC, major histocompatibility complex class I chain-related; GTN, glyceryl trinitrate.

**Table VII:** Expression levels of sMIC in serum.

Group	n	sMIC A ± SD, pg/ml	P-value	sMIC B ± SD, pg/ml	P-value
Normal	8	15.2±14.4	<0.001	2.5±10.2	<0.001
GTN	16	345.1±125.2		52.8±25.2	
Placebo	16	674.2±205.4		144.4±61.8	

P<0.01, GTN and placebo vs. normal. sMIC, soluble major histocompatibility complex class I chain-related; GTN, glyceryl trinitrate; SD, standard deviation.

**Table VIII:** Natural killer group 2 member D expression in the blood.

Group	n	Natural killer group 2 member D (mean ± standard deviation), %	P-value
Normal	8	68.51±19.42	<0.001
Glyceryl trinitrate	16	60.52±10.52	
Placebo	16	39.88±10.14	

P<0.01, GTN and normal vs. placebo.
